# Understanding treatment expectations toward emerging therapies in veterinary medicine: the role of knowledge, attitudes, and beliefs toward psychedelic-assisted interventions

**DOI:** 10.3389/fvets.2026.1864343

**Published:** 2026-07-28

**Authors:** Yanira Suárez-Fernández, Elisa Hernández-Álvarez, Lucas F. Borkel, Jaime Rojas-Hernández, Domingo J. Quintana- Hernández, Luis Alberto Henríquez-Hernández

**Affiliations:** 1 Faculty of Veterinary Medicine, Universidad de Las Palmas de Gran Canaria, Arucas, Spain; 2 Unit of Toxicology, Clinical Sciences Department, Universidad de Las Palmas de Gran Canaria, Las Palmas de Gran Canaria, Spain; 3 Universidad Fernando Pessoa Canarias, Guía, Spain; 4 Asociación Científica Psicodélica, Arucas, Spain; 5 Faculty of Psychology, Universidad del Atlántico Medio, Las Palmas de Gran Canaria, Spain

**Keywords:** animal welfare, psychedelic-assisted therapy, risk perception, treatment expectations, veterinary students

## Abstract

**Introduction:**

The adoption of emerging therapeutic approaches in veterinary medicine is likely to depend not only on scientific evidence but also on how such interventions are cognitively and attitudinally appraised by future professionals. This study aimed to assess knowledge, attitudes, and perceived risk regarding psychedelic use among veterinary students, and to examine their association with treatment expectations toward psychedelic-assisted therapies.

**Methods:**

A cross-sectional survey was conducted among undergraduate veterinary students (*N* = 87) at the University of Las Palmas de Gran Canaria during the 2025/2026 academic year. Data were collected using a structured questionnaire assessing knowledge and beliefs about psychedelics, alongside the Treatment Expectation Questionnaire (TEX-Q). Exploratory factor analysis identified two latent dimensions—positive attitudes and perceived risk—which were included as independent variables in subsequent analyses. Associations were examined using nonparametric tests, correlation analyses, and multiple linear regression models.

**Results:**

Positive attitudes showed the strongest and most consistent associations with favorable expectation domains, including perceived treatment benefit, impact, and overall expectations, while perceived risk showed an inverse, though more moderate, pattern of associations. Sociodemographic and educational variables were more closely associated with treatment expectations through these psychological constructs than through direct associations with the expectation domains themselves.

**Discussion:**

These findings are consistent with a cognitive–attitudinal framework in which expectations toward emerging therapies are more closely associated with interpretable psychological dimensions rather than with factual knowledge alone. Future longitudinal studies are warranted to clarify the relationships among these variables and to evaluate how educational experiences influence their development.

## 1 Introduction

Animal welfare is currently recognized as a central pillar of veterinary medicine, extending beyond diagnosis and treatment to encompass physical health, mental state, behavior, and overall quality of life. Contemporary veterinary practice increasingly incorporates a broader understanding of animals’ experiences, preferences, and environmental interactions, particularly in fields such as behavioral medicine, where anxiety and stress represent major clinical concerns (1, 2). International organizations emphasize the responsibility of veterinarians as key advocates for animal welfare and ethical decision-making (3, 4). Importantly, good welfare is directly associated with improved clinical outcomes, as reduced stress and pain facilitate recovery, enhance diagnostic accuracy, and improve anesthetic safety (3). Conversely, poor welfare—characterized by fear, pain, or distress—can lead to aggression, misdiagnosis, delayed healing, and long-term suffering (3, 5). However, beyond the classical framework of the Five Freedoms, animal welfare is increasingly understood as a complex and multifactorial construct (6). Its assessment is influenced not only by biological and environmental factors but also by human-related variables, including education and professional training (2, 7). This complexity is particularly evident in canine anxiety, a highly prevalent and often chronic condition associated with reduced quality of life, impaired human–animal relationships, and increased risk of relinquishment or euthanasia (8, 9). As a result, contemporary welfare science recognizes emotional states such as anxiety as central determinants of animal welfare.

Despite advances in behavioral and pharmacological management, current therapeutic approaches for anxiety-related conditions in dogs remain limited. Maladaptive anxiety is associated with dysregulation of neurochemical pathways involved in stress and emotional responses, which supports the use of pharmacological interventions targeting serotonergic and dopaminergic systems (10, 11). Commonly used treatments include antidepressants—such as selective serotonin reuptake inhibitors, tricyclic antidepressants, and monoamine oxidase inhibitors—as well as anxiolytics, including benzodiazepines and azapirones, administered either as monotherapy or in combination (12). However, these treatments show variable efficacy, often require individualized dose adjustments, and may be associated with adverse effects such as sedation, cardiovascular complications, tolerance, or even paradoxical worsening of symptoms (12, 13). In this context, the identification of novel therapeutic approaches has become a priority in both human and veterinary medicine. Among these, psychedelic-assisted interventions have gained increasing attention due to their potential to modulate key neurobiological pathways involved in emotional regulation, particularly through serotonergic mechanisms involving the 5-HT2A receptor (14–16). While emerging evidence suggests potential benefits in human psychiatric conditions (17, 18) and preliminary applications in canine anxiety (19, 20), their clinical use and translation into veterinary practice is still in its early stages. Recent experimental protocols have also explored the use of low-dose lysergic acid derivatives in dogs with chronic osteoarthritic pain (21), a condition closely linked to impaired welfare (22), suggesting a potential broader therapeutic scope beyond behavioral disorders. Within controlled settings, these approaches have shown a favorable safety profile, with no relevant adverse effects have been reported in controlled experimental settings to date (23–25). However, their clinical use remains highly restricted, and further research is needed to establish their efficacy, safety, and applicability in veterinary contexts.

The adoption of emerging therapeutic approaches in veterinary medicine is influenced not only by the available scientific evidence but also by the cognitive and attitudinal framework through which professionals interpret that evidence (26, 27). Factors such as perceived knowledge, beliefs about efficacy and safety, and risk perception play a central role in shaping clinical decision-making and openness to innovation (28, 29). In this context, veterinary students represent a particularly relevant population, as their perceptions and expectations may influence future prescribing behaviors and the integration of novel therapies into clinical practice. Recent evidence from health sciences student population supports this perspective. A cross-sectional study conducted among medical and nursing students showed that attitudes toward psychedelic-assisted therapies are significantly influenced by sociodemographic variables, prior experience, and formal education, with training associated with greater knowledge and more favorable attitudes toward therapeutic use (30). These findings suggest that educational exposure may play a key role in reducing stigma and fostering evidence-based perspectives on emerging treatments. Understanding how these cognitive and attitudinal variables interact is therefore essential to anticipate potential barriers and facilitators in the implementation of novel therapeutic strategies.

Within this framework, treatment expectations emerge as a key psychological construct influencing the evaluation and potential adoption of novel therapeutic approaches (31). Expectations encompass beliefs regarding treatment efficacy, risks, impact on quality of life, and overall outcomes, and have been shown to play a significant role in clinical decision-making and therapeutic processes (32). Evidence from human healthcare indicates that clinicians often hold inaccurate expectations regarding the benefits and harms of interventions, frequently overestimating benefits while underestimating risks, which may lead to suboptimal clinical decisions (33). However, despite their relevance, validated instruments specifically designed to assess treatment expectations in veterinary contexts remain scarce. In the context of emerging interventions, such as psychedelic-assisted therapies, expectations may be particularly sensitive to underlying attitudes, perceived knowledge, and risk appraisal (34, 35). Therefore, examining treatment expectations in veterinary students provides a relevant approach to understanding how cognitive and attitudinal factors may shape the future integration of these therapies into clinical practice.

Accordingly, the aim of this study was to assess knowledge, attitudes, and perceived risk regarding psychedelic use among veterinary students, and to examine their association with treatment expectations toward psychedelic-assisted therapies. In addition, the study sought to explore the associations of sociodemographic, educational, and cognitive factors with different domains of treatment expectations, thereby providing a conceptual basis for understanding how expectations toward emerging therapeutic approaches are formed during veterinary training.

## 2 Materials and methods

### 2.1 Study design and population

This study was designed as a cross-sectional observational survey conducted among undergraduate students enrolled in the Veterinary Medicine degree program at the University of Las Palmas de Gran Canaria (Canary Islands, Spain) during the 2025/2026 academic year. The target population included students from all academic years, with the aim of assessing knowledge, perceptions, and expectations regarding the potential use of psychedelic-assisted therapies in veterinary clinical practice. No exclusion criteria were applied. Participation was voluntary and anonymous, with no collection of personally identifiable information. The study was framed within an exploratory design with confirmatory components, given the emerging nature of psychedelic research in veterinary medicine and the limited availability of prior validated instruments in this population.

According to official data provided by the Dean’s Office of the Faculty of Veterinary Medicine, a total of 375 students were enrolled in the program during the study period, distributed across academic years as follows: 77 in the first year, 75 in the second year, 80 in the third year, 78 in the fourth year, and 65 in the fifth year. All students were invited to participate in the study through official electronic communication from the Dean’s Office and via dissemination of the survey link through institutional channels and student communication platforms.

The questionnaire began with an introductory section describing the objectives of the study, as well as assurances regarding anonymity, confidentiality, and data protection. To ensure participant anonymity, IP address tracking, cookies, and any identifying metadata were disabled. Participation was entirely voluntary, with no incentives or penalties associated with participation or non-participation. Informed consent was obtained implicitly through voluntary agreement to proceed with the questionnaire. The study was approved by the Research Ethics Committee of the province of Las Palmas, Spain (ethical approval code #2025-468-1). Due to the fully anonymous nature of data collection, the Research Ethics Committee waived the requirement for written informed consent. All procedures were conducted in accordance with the ethical standards for research involving human participants and complied with the principles of the Declaration of Helsinki.

### 2.2 Data collection instruments

Two structured questionnaires were used for data collection. In addition to attitudinal and perception-based items, the survey collected sociodemographic and educational information, including age, gender, academic year, prior personal experience with psychedelic substances, and prior academic training related to psychedelics. Substances included in the assessment of prior experience comprised MDMA (ecstasy), psilocybin (magic mushrooms), and/or lysergic acid diethylamide (LSD), which are among the most widely studied compounds in current psychedelic-assisted therapy research. With respect to formal education, structured curricular content on psychedelics within the Veterinary Medicine program is limited but integrated in a transversal manner within the Toxicology course (7th semester), where foundational concepts related to psychoactive substances, including psychedelics, are addressed in a veterinary clinical context. No mandatory standalone course specifically dedicated to psychedelics is included in the curriculum. In addition, students are offered the opportunity to voluntarily enroll in an interdisciplinary elective course on psychedelic-assisted therapies provided by the institution. It is acknowledged that additional knowledge or exposure may have been acquired outside the formal academic program through personal interest, independent study, or extracurricular activities. Such sources of informal learning may have influenced participants’ self-perceived knowledge and attitudinal responses toward psychedelic substances.

The final questionnaire was administered using the Google Surveys platform. Given the nature of digital dissemination, complete control over secondary sharing of the survey link could not be guaranteed. The data collection period extended from 18 November 2025 to 15 March 2026.

#### 2.2.1 Expectation of treatment outcomes scale (TEX-Q)

A previously validated multidimensional scale was used to assess expectations regarding treatment outcomes. The instrument consisted of 15 items rated on a 10-point Likert-type scale (0 = “not at all” to 10 = “extremely”). The scale evaluates multiple domains of treatment expectations, including perceived benefits, anticipated risks, functional impact, and quality of life. The instrument was originally developed and validated as the Treatment Expectation Questionnaire (TEX-Q), a generic multidimensional measure of patient treatment expectations (31). Although the TEX-Q was not originally developed for veterinary populations, it was considered appropriate for this study because it assesses general dimensions of treatment expectations rather than disease-specific experiences. Nevertheless, its use in veterinary student populations should be interpreted as an extension of its application rather than a revalidation of the instrument. The Spanish version used in the present study was based on a previously applied adaptation in veterinary research involving canine behavioral and welfare outcomes (24). For this study, the survey items were translated from English into Spanish following a forward and backward translation procedure to ensure conceptual, semantic, and linguistic equivalence. The translation was initially performed by one of the co-authors and subsequently reviewed by the research team to ensure consistency with the original instrument. The Spanish-translated version was used for data collection, and all items were subsequently aggregated into six theoretically derived dimensions, calculated as the mean of the corresponding items within each domain, following the structure proposed in the original validation study (31). Generative artificial intelligence (ChatGPT, OpenAI, GPT-4.1-mini model) was used exclusively as a linguistic support tool for grammar and scientific English refinement during manuscript preparation. The authors take full responsibility for the content, interpretation, and scientific integrity of the work. The final Spanish version of the instrument is provided in Appendix A to facilitate reproducibility and future research.

#### 2.2.2 Questionnaire on knowledge, perceptions, and beliefs regarding psychedelic use

A structured questionnaire consisting of 7 items was used to assess veterinary students’ knowledge, perceptions, and beliefs regarding the use of psychedelic substances in humans and their potential therapeutic applications. The instrument is based on a previously published questionnaire developed for assessing attitudes toward psychedelic-assisted therapies in medical student populations (36), and the same item structure was used in the present study without modification of item content. The only adaptation performed was linguistic translation into Spanish to ensure comprehension in the target population, as previously reported (30).

Items were rated on a 5-point Likert scale (1 = “strongly disagree” to 5 = “strongly agree”), covering domains related to perceived knowledge, therapeutic potential, perceived risks, and general beliefs about psychedelic use. Given that the instrument has not been formally validated in veterinary student populations and considering the absence of an established factorial structure in this context, an exploratory factor analysis (EFA) was conducted to examine its underlying dimensionality and to identify latent constructs emerging from the data. The resulting factor structure was used to derive composite variables representing coherent psychological dimensions related to knowledge, attitudes, and risk perception regarding psychedelic substances.

### 2.3 Statistical analysis

Descriptive analyses were performed for all study variables. Continuous variables were summarized using measures of central tendency and dispersion, while categorical variables were described using frequencies and percentages.

Inferential analyses were conducted to examine associations between sociodemographic characteristics, knowledge and belief constructs, attitudinal dimensions, and treatment expectation domains. Normality was assessed using the Kolmogorov–Smirnov test complemented by visual inspection of distributional plots. Since continuous variables did not meet normality assumptions, group comparisons were performed using nonparametric tests, specifically the Mann–Whitney U test for two-group comparisons. Associations between categorical variables were assessed using the Chi-square test, with Fisher’s exact test applied when expected cell counts were below five to ensure statistical validity. Relationships between continuous or ordinal variables were evaluated using correlation analyses. Spearman’s rank correlation coefficient was used for ordinal or non-normally distributed variables, while Pearson’s correlation coefficient was applied to composite variables exhibiting approximately continuous behavior, such as aggregated scale scores derived from multiple items. This analytical strategy was designed to explore the relationships between cognitive, attitudinal, and experiential variables and their contribution to treatment expectations in veterinary students, as well as to identify their independent predictive value across different expectation domains.

In addition, exploratory comparisons were conducted using individual-level data from our previously published study of medical and nursing students (30). These data had been collected by the same research group using the same questionnaire and comparable survey procedures, allowing direct comparison with the veterinary student cohort. Because the original datasets were available, all three degree programs (Veterinary Medicine, Medicine, and Nursing) were analyzed within a common statistical framework. These analyses were considered exploratory and were included solely to provide broader educational context rather than to test a primary study hypothesis. Given the approximate continuous behavior of aggregated Likert-scale scores and their suitability for parametric approximation at the composite level, one-way analysis of variance (ANOVA) was used to assess overall differences between groups. When statistically significant effects were detected, *post hoc* pairwise comparisons were performed using Tukey’s Honestly Significant Difference (HSD) test to identify specific group differences.

Multiple linear regression analyses were conducted to assess the independent contribution of sociodemographic, educational, and psychological variables (knowledge, attitudes, and perceived risk) to each treatment expectation domain. Separate models were fitted for each dependent variable using the enter method, with all independent variables entered simultaneously. All statistical tests were two-tailed, and the significance level was set at *p* < 0.05. Analyses were conducted using IBM SPSS Statistics (version 19.0, IBM Corp., Armonk, NY, USA).

#### 2.3.1 Exploratory factor analysis and construction of latent dimensions

An exploratory factor analysis (EFA) was conducted to examine the underlying structure of the 7-item questionnaire assessing knowledge, perceptions, and beliefs regarding psychedelic use. The adequacy of the data for factor analysis was assessed using the Kaiser–Meyer–Olkin (KMO) measure and Bartlett’s test of sphericity. The KMO value was 0.717, indicating an acceptable level of sampling adequacy. Bartlett’s test of sphericity was statistically significant (χ^2^ = 218.459; df = 28; *p* < 0.001), confirming that the correlation matrix was suitable for factor analysis.

The EFA was performed using principal component analysis as the extraction method, followed by Varimax rotation with Kaiser normalization to improve interpretability of the resulting factor structure. Two factors with eigenvalues greater than 1 were identified, explaining the underlying dimensional structure of the instrument. Based on the rotated component matrix, a bidimensional structure emerged. The first factor comprised items reflecting positive attitudes toward the therapeutic use of psychedelics (items 6, 7, and 8, together with item 5 reverse-coded). The second factor reflected perceived risks associated with psychedelic use (items 2 and 3). Item Q1 (self-perceived knowledge) and item Q4 (belief regarding legality of recreational use) did not load clearly onto either factor and were therefore retained as independent variables for subsequent analyses. Latent dimensions were constructed by calculating the arithmetic mean of the items loading on each factor. Items with negative wording were reverse coded prior to aggregation to ensure directional consistency across scales. Only items with factor loadings ≥ 0.40 were considered for inclusion in the corresponding composite variables.

#### 2.3.2 Reliability analysis

Internal consistency of the derived dimensions was assessed using Cronbach’s alpha coefficient. The “positive attitudes toward psychedelic therapy” dimension showed good internal consistency (*α* = 0.803). The “perceived risk” dimension also demonstrated adequate internal reliability (α = 0.804). Given that the latter consisted of only two items, internal consistency was additionally assessed using Pearson’s correlation coefficient between both items, which showed a strong and statistically significant association (*r* = 0.691; *p* < 0.001), supporting the coherence of the construct.

## 3 Results

A total of 87 students completed the survey. Descriptive characteristics of the sample are presented in Table 1. The median age was 22 years (interquartile range [IQR]: 20–24), the majority of participants were female (n = 69, 79.3%) and enrolled in the fourth or fifth year of the Veterinary Medicine degree at the University of Las Palmas de Gran Canaria (n = 53, 60.9%). Only seven participants (8.0%) reported previous experience with psychedelics, specifically referring to MDMA, psilocybin, and/or LSD. In that sense, previous experience with psychedelics was significantly higher among male students (22.2%) compared to female students (4.3%). Given the small number of cases, these differences should be interpreted with caution (Fisher exact test, *p* = 0.026). A total of 48 respondents (55.2%) reported having received formal academic training on psychedelics.

**Table 1:** Descriptive analysis of the study sample (*n* = 87).

Variable	*N* (%) or median (IQR)
Age (years)^1^	22 (24–20)
Gender	
Female	69 (79.3)
Male	18 (20.7)
Academic year	
1–3	34 (39.1)
4–5	53 (60.9)
Previous experience with psychedelics^2^	
No	80 (92.0)
Yes	7 (8.0)
Academic training on psychedelics	
No	39 (44.8)
Yes	48 (55.2)

IQR, interquartile range.

^1^Non-normal distribution (Kolmogorov–Smirnov test; *p* value < 0.001).

^2^Refers to MDMA, psilocybin, or LSD.

The attitudes and beliefs questionnaire on psychedelics consisted of eight items. The full frequency distribution of responses is presented in Supplementary Table 1. The distribution of responses showed a heterogeneous pattern. Most participants did not consider themselves well-informed about psychedelics (mean = 2.6), while a large proportion perceived substantial risks, particularly in relation to cognitive impairment and psychiatric outcomes (mean = 4.2 and 3.9, respectively). In contrast, most respondents disagreed with the statement that psychedelic use is unsafe under medical supervision (mean = 2.0) and reported high levels of agreement with the need for further research (mean = 4.3).

Exploratory comparisons across degree programs revealed significant differences in several attitudinal items (Supplementary Table 2). Overall, veterinary students showed a more cautious profile compared to nursing students, particularly regarding perceived safety and therapeutic potential, while medical students tended to occupy an intermediate position between both groups. No substantial differences were observed between veterinary and medical students in self-perceived knowledge.

The Treatment Expectations Questionnaire (TEX-Q) consisted of 15 items. The full frequency distribution of responses is presented in Supplementary Table 3. Overall, responses to the TEX-Q showed higher scores in items related to expected treatment benefit, positive impact, and process (Q1–Q6 and Q12–Q15), with mean values ranging from 6.06 (Q6, perceived improvement in the animal’s ability to adapt to environmental demands) to 7.24 (Q15, perceived influence of the veterinarian’s own behavior on treatment success). In contrast, items reflecting negative expectations, including perceived risks and adverse effects (Q7–Q11), showed lower or more variable scores (mean = 4.97 and 5.00, respectively).

To facilitate a more comprehensive analysis, items from the attitudes and beliefs scale were grouped into four dimensions. Items 1 (I consider myself well-informed about psychedelics) and 4 (Recreational use of psychedelics should remain illegal) were retained as individual variables, as they were not included in the factor structure derived from the exploratory factor analysis. The remaining items were organized into two factors: “positive attitudes toward the therapeutic use of psychedelics” (calculated as the mean of items 5–8) and “perceived risk” (calculated as the mean of items 2 and 3). Similarly, the 15 items of the TEX-Q were grouped into seven dimensions, as previously described (31) and detailed in the Methods section: treatment benefit, positive impact, adverse events, negative impact, process, behavioral control, and total score. Table 2 summarizes the main results derived from these aggregated variables. On a 5-point scale, the mean score for agreement with the illegality of recreational psychedelic use was 3.7. The mean scores for positive attitudes toward therapeutic use and perceived risk were 3.9 and 4.1, respectively. Regarding treatment expectations, the highest mean scores were observed for behavioral control (6.8) and treatment benefit (6.6) on a 0–10 scale. In contrast, lower mean values were found for negative expectation domains, with negative impact showing a mean score of 3.3, indicating a relatively low expectation of negative effects associated with the treatment. The overall expectation score had a mean value of 6.4 (Table 2).

**Table 2:** Descriptive statistics of attitudes, beliefs, and treatment expectations toward psychedelic use among veterinary students.

Item	Mean	SD
Attitudes and beliefs questionnaire*		
I consider myself well-informed about psychedelics	2.6	1.1
Recreational use of psychedelics should remain illegal	3.7	1.1
Positive attitudes toward therapeutic use^1^	3.9	0.7
Perceived risk associated with psychedelic use^2^	4.1	0.7
TEX-Q**		
Treatment benefit	6.6	2.0
Positive impact	6.5	2.1
Adverse events	4.5	2.2
Negative impact	3.3	2.3
Process	6.3	2.1
Behavioral control	6.8	2.0
Total score	6.4	1.8

Values are presented as mean and standard deviation (SD).

*Items were rated on a 5-point Likert scale (1–5).

**Items were rated on a 0–10 scale.

^1^Mean score of four items assessing positive attitudes toward therapeutic use: Q5 (inverted), The use of psychedelics is unsafe even under medical supervision; Q6, Psychedelic use has shown promising results in the treatment of psychiatric disorders; Q7, Psychedelic use may enhance treatment outcomes when used in conjunction with psychotherapy; and Q8, Psychedelic use warrants further investigation as a potential treatment for psychiatric disorders.

^2^Mean score of two items assessing perceived risk: Q2, The use of psychedelics increases the risk for subsequent psychiatric disorders; and Q3, The use of psychedelics increases the risk of long-term cognitive impairment.

Several sociodemographic, experiential, and cognitive variables were significantly associated with different domains of perceptions, beliefs, and treatment expectations. A significant negative correlation was observed between age and expected treatment benefit (Pearson’s *r* = −0.246, *p* = 0.021). No significant differences were found according to gender or previous psychedelic experience in the main expectation domains. However, male participants reported higher self-perceived knowledge about psychedelics than females (χ^2^ test, *p* = 0.023) and were less likely to support the maintenance of prohibition in recreational contexts (χ^2^ test, *p* = 0.032). Moreover, the proportion of respondents opposing the maintenance of illegality was significantly higher among those reporting previous psychedelic experience (χ^2^ test, *p* = 0.020). Participants reporting previous academic training on psychedelics were also characterized by higher positive attitude scores (4.0 vs. 3.7, *p* = 0.040, Mann–Whitney U test), as well as higher expected treatment benefit scores (6.8 vs. 6.2, *p* = 0.027) and more favorable expectations regarding the therapeutic process (6.9 vs. 5.7, *p* = 0.005). No significant association was observed between academic training and academic year.

A comprehensive overview of the correlations between attitudinal variables and treatment expectation domains is presented in Table 3.

**Table 3:** Pearson correlation matrix among study variables.

Item	Info	Illegal	Attitude	Risk	Benefit	Impact	Adverse	Negative	Process	Control	Total
Info	1										
*p*-value											
Illegal	**−0.213**	1									
*p*-value	0.048										
Attitude	**0.354**	−0.209	1								
*p*-value	0.001	0.052									
Risk	0.200	0.187	0.078	1							
*p*-value	0.063	0.083	0.473								
Benefit	**0.225**	−0.093	**0.613**	−0.039	1						
*p*-value	0.036	0.392	0.000	0.719							
Impact	0.155	−0.083	**0.593**	−0.072	**0.917**	1					
*p*-value	0.151	0.447	0.000	0.508	0.000						
Adverse	−0.049	−0.120	**−0.345**	0.132	**−0.541**	**−0.566**	1				
*p*-value	0.653	0.267	0.001	0.224	0.000	0.000					
Negative	−0.060	−0.064	**−0.468**	0.168	**−0.580**	**−0.618**	**0.834**	1			
*p*-value	0.584	0.557	0.000	0.120	0.000	0.000	0.000				
Process	0.152	0.051	**0.381**	−0.006	**0.614**	**0.631**	**−0.618**	**−0.601**	1		
*p*-value	0.159	0.642	0.000	0.954	0.000	0.000	0.000	0.000			
Control	0.066	−0.084	**0.359**	−0.030	**0.631**	**0.664**	**−0.462**	**−0.504**	**0.581**	1	
*p*-value	0.544	0.439	0.001	0.782	0.000	0.000	0.000	0.000	0.000		
Total	0.145	−0.005	**0.562**	−0.093	**0.873**	**0.895**	**−0.820**	**−0.833**	**0.796**	**0.749**	1
*p*-value	0.181	0.966	0.000	0.394	0.000	0.000	0.000	0.000	0.000	0.000	

Correlation coefficients (*r*) and corresponding *p*-values are presented. Statistically significant correlations (*p* < 0.05) are highlighted in bold. All tests are two-tailed. Attitudes and beliefs questionnaire: Info, I consider myself well-informed about psychedelics; Illegal, Recreational use of psychedelics should remain illegal; Attitude, Positive attitudes toward therapeutic use; Risk, Perceived risk associated with psychedelic use.

TEX-Q: Benefit, Treatment benefit; Impact, Positive impact; Adverse, Adverse events; Negative, Negative impact; Process; Control, Behavioral control; Total, Total score.

Self-perceived knowledge about psychedelics was positively correlated with positive attitudes toward their therapeutic use (*r* = 0.354, *p* = 0.001) and with expected treatment benefit (*r* = 0.225, *p* = 0.036). A weak but significant negative correlation was observed between self-perceived knowledge and support for the illegality of psychedelics (*r* = −0.213, *p* = 0.048).

Positive attitudes toward the therapeutic use of psychedelics were significantly associated with several treatment expectation domains. Specifically, positive correlations were observed with treatment benefit (*r* = 0.613, *p* < 0.001), positive impact (*r* = 0.593, *p* < 0.001), process (*r* = 0.381, *p* < 0.001), behavioral control (*r* = 0.359, *p* = 0.001), and total score (*r* = 0.562, *p* < 0.001). Conversely, positive attitudes were negatively correlated with expectations of adverse events (*r* = −0.345, *p* = 0.001) and negative impact (*r* = −0.468, *p* < 0.001).

To further examine the independent contribution of each variable to treatment expectations, a series of multivariable linear regression models were conducted. These models were chosen to account for the simultaneous influence of sociodemographic, experiential, and cognitive factors on each expectation domain, while controlling for potential confounding effects. Separate models were fitted for each TEX-Q domain as dependent variables. A full model approach (enter method) was applied, with all prespecified independent variables entered simultaneously into each model. Detailed results for each model are provided in Table 4.

**Table 4:** Model fit statistics for multiple linear regression analyses of treatment expectation domains.

Model	R^2^	Adjusted R^2^	*F*	*p* (ANOVA)
Benefit	0.547	0.494	10.330	<0.001
Impact	0.487	0.427	8.135	<0.001
Adverse	0.272	0.187	3.204	0.002
Negative	0.368	0.294	4.985	<0.001
Process	0.271	0.186	3.188	0.003
Control	0.190	0.095	2.001	0.050
Total	0.457	0.394	7.215	<0.001

Coefficients of determination (R^2^), adjusted R^2^, F-statistics, and corresponding *p*-values from ANOVA are presented for each model.

R^2^, coefficient of determination; Adjusted R^2^, adjusted coefficient of determination.

All models were estimated using multiple linear regression.

No multicollinearity was detected (all variance inflation factor [VIF] values < 2.5).

The regression models accounted for different proportions of variance across the TEX-Q domains. The largest proportions of explained variance were observed for treatment benefit (R^2^ = 0.547) and positive impact (R^2^ = 0.487), followed by the total score (R^2^ = 0.457). Intermediate proportions of explained variance were observed for negative impact (R^2^ = 0.368), adverse events (R^2^ = 0.272), and process (R^2^ = 0.271). In contrast, the behavioral control domain showed a lower level of explained variance (R^2^ = 0.190; *p* = 0.050).

The associations between the independent variables and the treatment expectation domains are presented in Table 5, which summarizes the standardized regression coefficients (*β*) derived from the multivariable models. Positive attitudes toward the therapeutic use of psychedelics showed the strongest and most consistent associations across the models. Higher levels of positive attitudes were associated with greater expected treatment benefit (*β* = 0.614), positive impact (*β* = 0.616), process (*β* = 0.385), behavioral control (*β* = 0.407), and overall expectations (*β* = 0.597), while being inversely associated with expectations of adverse events (*β* = −0.396) and negative impact (*β* = −0.518). Perceived risk also showed a consistent, although generally more moderate, pattern of associations. Higher perceived risk was associated with lower expected benefit (*β* = −0.178), impact (*β* = −0.183), and overall expectations (*β* = −0.215), and with higher expectations of adverse events (*β* = 0.235) and negative impact (*β* = 0.251).

**Table 5:** Standardized regression coefficients (*β*) from multiple linear regression models examining the associations between study variables and treatment expectation domains.

Variable	TEX-Q						
	Benefit	Impact	Adverse	Negative	Process	Control	Total
Gender	−0.216*	−0.188*	ns	ns	ns	ns	−0.200*
Age	−0.267**	−0.213*	0.214*	0.196*	ns	ns	−0.248**
Academic year	ns	ns	ns	ns	ns	ns	ns
Experience	ns	ns	ns	ns	ns	ns	ns
Training	ns	ns	ns	ns	0.269*	ns	ns
Knowledge	ns	ns	ns	ns	ns	ns	ns
Illegality	ns	ns	−0.269*	−0.252*	ns	ns	ns
Positive attitudes	0.614***	0.616***	−0.396***	−0.518***	0.385**	0.407***	0.597***
Perceived risk	−0.178*	−0.183*	0.235*	0.251*	ns	ns	−0.215*

Each column represents a separate dependent variable. Only standardized coefficients are shown for clarity. Statistical significance is indicated as follows: *p < 0.05, **p < 0.01, ***p < 0.001. Non-significant associations are marked as “ns”.

All models were estimated using multiple linear regression. No multicollinearity was detected in any model (all variance inflation factor (VIF) values < 2.5).

Age was treated as a continuous variable; academic year as a dichotomous variable (1–3 vs. 4–5).

Among sociodemographic variables, age and gender showed selective associations. Older age was associated with lower expected benefit (*β* = −0.267), impact (*β* = −0.213), and total scores (*β* = −0.248), but with higher expectations of adverse events (*β* = 0.214) and negative impact (*β* = 0.196). Compared to females, male participants showed lower expected benefit (*β* = −0.216), impact (*β* = −0.188), and overall expectations (*β* = −0.200). Academic training was positively associated with expectations related to the therapeutic process (*β* = 0.269), while beliefs regarding the illegality of psychedelics were associated with lower expectations of adverse events (*β* = −0.269) and negative impact (*β* = −0.252). No significant independent associations were observed for academic year, previous psychedelic experience, or self-perceived knowledge.

## 4 Discussion

Overall, the findings are consistent with a coherent cognitive–attitudinal framework in which treatment expectations toward psychedelic-assisted therapies are closely associated with two central psychological constructs: positive attitudes and perceived risk. This pattern is consistent with broader models of clinical decision-making and innovation adoption, which propose that judgments about novel interventions are rarely associated with factual knowledge alone but instead reflect affective and cognitive appraisal processes through which perceived benefits and harms are interpreted (26, 28, 32, 33). Although this cognitive–attitudinal framework provides a coherent interpretation of the present findings, it should be understood within the context of the study population. The participants were veterinary students from a single institution whose expectations are likely to reflect an educational rather than a clinical perspective. Consequently, these findings should not be assumed to generalize directly to veterinary students from other educational settings or cultural contexts, nor to practicing veterinarians, whose judgments whose judgments are likely to reflect the combined influence of clinical experience, professional responsibilities, and greater reliance on evidence-based decision-making (37, 38). Future studies including diverse educational institutions and experienced veterinary professionals will be important to determine the extent to which the proposed model applies across different stages of veterinary training and professional practice. Moreover, because the present study employed a cross-sectional design, the proposed relationships should be interpreted as associative rather than causal. Longitudinal research will be necessary to determine how knowledge, attitudes, perceived risk, and treatment expectations evolve over time, and whether educational experiences contribute to changes in these cognitive–attitudinal processes.

**Figure 1:**
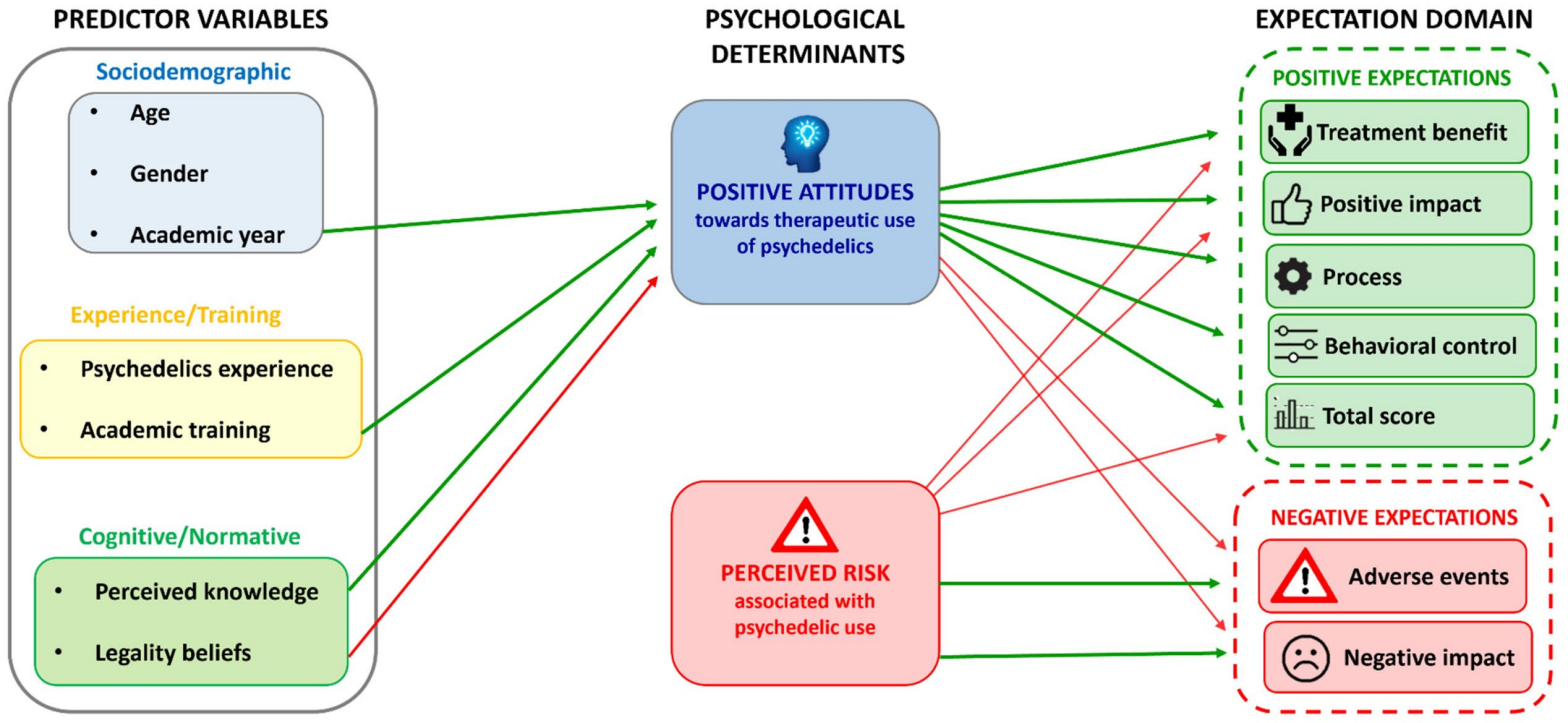
Proposed conceptual framework of the observed associations underlying expectations toward psychedelic-assisted therapies. Arrows represent statistically significant associations. Green arrows indicate positive associations, whereas red arrows indicate negative associations. Associations between the independent variables and the psychological constructs (positive attitudes and perceived risk) are based on bivariate analyses (Mann–Whitney U test, Spearman or Pearson correlation), whereas associations between the psychological constructs and expectation domains are derived from multivariate linear regression models (see Table 5). Only significant relationships are shown. The direction of the arrows reflects the analytical sequence used to organize the findings and should not be interpreted as evidence of causal relationships. The framework is intended as an interpretative, hypothesis-generating synthesis of the observed associations.

As illustrated in Figure 1, the observed pattern of associations can be summarized within a conceptual framework linking sociodemographic characteristics, prior experience, academic training, and cognitive factors such as perceived knowledge and legality beliefs with the different treatment expectation domains assessed by the TEX-Q. Positive attitudes showed the strongest and most consistent associations across the models, being positively associated with favorable expectation domains and inversely associated with negative expectations. In contrast, perceived risk showed an opposite, although generally more moderate, pattern of associations. Together, these findings suggest that expectations toward emerging therapies in veterinary contexts are more closely associated with higher-order cognitive–attitudinal constructs than with background characteristics alone. This conceptual framework is consistent with the view that treatment expectations may be understood as emergent cognitive constructs rather than direct reflections of informational exposure (39).

These cognitive–attitudinal structures may be particularly sensitive in fields such as psychedelic research, where scientific development has historically been intertwined with experiential knowledge. Evidence indicates that personal psychedelic use is highly prevalent among researchers in the field, with reports suggesting that up to 85% have experienced classic psychedelics (40). Such experiences are frequently described as a key factor in shaping initial interest in psychedelic science and in facilitating engagement with phenomenological and therapeutic dimensions of these substances (40). Moreover, personal use has been associated with more positive evaluations of the potential impact of psychedelics on well-being, psychological health, and broader societal domains, with effect sizes in the moderate range (40). While experiential exposure may enhance insight into subjective and clinical processes, it also raises important concerns regarding potential biases in scientific interpretation. Commentaries in the field have highlighted that psychedelic experiences may induce heightened enthusiasm even among otherwise methodologically conservative researchers, potentially influencing expectations, reducing critical distance, and increasing vulnerability to interpretative bias (41, 42). Historical examples have further illustrated how strong experiential engagement with psychedelics has, in some cases, blurred boundaries between scientific inquiry and advocacy, underscoring the need for explicit safeguards such as conflict-of-interest policies and reflexive methodological standards to preserve scientific rigor (41, 42). This dual role of experience—as both epistemic resource and potential source of bias—highlights the importance of carefully contextualizing attitudes and expectations within emerging therapeutic domains. Within this framework, perceived risk and positive attitudes identified in the present study may be interpreted as downstream cognitive expressions of this broader tension between experiential salience and evidential appraisal.

If such epistemic processes operate even among experienced researchers (42), similar mechanisms are likely to be amplified in populations still undergoing formal training, where attitudes and expectations are likely to reflect institutional curricula and early professional socialization (43). The observed associations may also reflect differences in students’ educational exposure to psychedelic-related topics. Although this variability could have contributed to differences in perceived knowledge, attitudes, and treatment expectations, it likely reflects the current educational reality, in which formal instruction on these therapies remains limited and non-standardized rather than a source of experimental bias. In this regard, the present findings are consistent with the notion that educational context may contribute to structuring cognitive–attitudinal responses toward emerging therapies (44, 45). Exploratory comparisons across degree programs revealed a consistent pattern of differences in key attitudinal domains, particularly those related to perceived safety, therapeutic efficacy, and openness to further research. Overall, veterinary students tended to exhibit a more cautious appraisal of psychedelic-assisted therapies compared to their counterparts in nursing and, to a lesser extent, medicine (30). Notably, this pattern was not driven by differences in self-perceived knowledge, which was comparable between veterinary and medical students, suggesting that the observed variability is unlikely to reflect purely informational exposure (39, 43). Instead, it may indicate differences in how disciplinary training frames uncertainty, risk, and pharmacological innovation. These findings are consistent with the proposed cognitive–attitudinal framework by suggesting that educational background may be associated with how perceived knowledge is related to attitudes and risk–benefit appraisals (34), rather than being directly associated with treatment expectations. This supports the notion that expectations toward novel therapeutic modalities are not merely knowledge-dependent, but are filtered through discipline-specific epistemic frameworks that organize the interpretation of uncertainty and clinical innovation (28, 34, 39, 43). In veterinary education specifically, this may be particularly relevant given the relative scarcity of structured exposure to emerging psychopharmacological paradigms, which may accentuate reliance on generalized risk heuristics rather than evidence-weighted appraisal of therapeutic potential.

Within this context, the present findings extend current understanding of how treatment expectations are constructed in non-clinical populations by suggesting that they are associated not only with knowledge acquisition (46), but also by stable attitudinal and risk-related dimensions that appear to organize cognitive appraisal across different domains of anticipated therapeutic outcomes (47). This pattern is consistent with the possibility that expectations function as structured psychological representations of uncertainty, integrating informational, experiential, and normative inputs into coherent evaluative judgments. From an applied perspective, this has direct implications for veterinary medical education. If the observed associations reflect modifiable cognitive constructs, then educational interventions may benefit from explicitly targeting not only knowledge gaps, but also underlying evaluative frameworks such as risk perception, uncertainty tolerance, and attitude formation (Figure 1). In this sense, traditional didactic approaches may be insufficient to modify expectations in a meaningful way unless they also address how students interpret and integrate ambiguous or emerging scientific evidence (48, 49). Finally, these results highlight the potential value of incorporating cognitive–attitudinal perspectives into the study of innovation adoption in veterinary medicine. As new therapeutic modalities continue to emerge in areas such as behavioral pharmacology and psychopharmacological adjuncts, understanding how expectations are associated with cognitive–attitudinal processes at early stages of professional development may be critical for ensuring that future clinical decision-making remains both evidence-informed and appropriately calibrated to uncertainty (47, 49). This may be particularly important in welfare-relevant conditions such as canine anxiety and chronic behavioral disorders, where therapeutic innovation is both needed and methodologically challenging.

This study presents several limitations. First, the sample size (n = 87) and its restriction to a single veterinary school limit the generalizability of the findings to other institutions and cultural contexts. Although the target population was fully accessible, participation was voluntary, which may have introduced self-selection bias, with potential overrepresentation of students who were more interested in emerging or unconventional therapeutic approaches. In addition, female students accounted for a larger proportion of respondents. While this distribution broadly reflects the female predominance commonly observed in veterinary degree programs (50), it remains possible that sex-related differences in willingness to participate may have influenced the sample composition. Because participants’ motivations for taking part in the survey were not assessed, the extent to which self-selection may have affected the representativeness of the findings cannot be determined. Second, the cross-sectional design precludes causal inferences regarding the relationships between cognitive–attitudinal constructs and treatment expectations. Although the analyses identified meaningful associations consistent with the proposed conceptual framework, the causality of these relationships cannot be established. Longitudinal studies will be necessary to determine how knowledge, attitudes, perceived risk, and treatment expectations evolve throughout veterinary training, and whether educational experiences contribute to changes in these constructs. Third, the use of self-reported measures may have introduced social desirability and response biases, which could have influenced participants’ responses despite the anonymous nature of the survey. Although validated and structured instruments were used where available, part of the assessment relied on an instrument that had not previously been validated in veterinary students. Consequently, the exploratory factor analysis should be regarded as a preliminary examination of its internal structure rather than a definitive psychometric validation. Replication in larger samples using confirmatory factor analysis will be necessary to establish the robustness and generalizability of the proposed factor structure. Moreover, the use of the TEX-Q in a veterinary student population builds on previous adaptations of the instrument in non-clinical contexts and should be interpreted as an extension of its application rather than a revalidation in this specific population. Fourth, although group comparisons were appropriately conducted using non-parametric or parametric tests depending on distributional assumptions, the large number of exploratory comparisons increases the risk of type I error, and results should therefore be interpreted with appropriate caution. Accordingly, the observed associations should be considered hypothesis-generating and require replication in larger, independent, and more diverse samples before broader generalizations can be made. Fifth, the perceived risk construct was represented by only two items. Although these items demonstrated good internal consistency and a strong inter-item correlation, future studies should seek to confirm this construct using more comprehensive instruments with additional indicators. Finally, an additional limitation concerns the heterogeneity of formal exposure to psychedelic-related topics within the curriculum. Although some content is addressed transversally in Toxicology teaching, no structured or standardized training exists across all students. Consequently, differences in educational experience may have influenced perceived knowledge, attitudes, and treatment expectations. However, this variability reflects the current educational reality rather than an experimental condition and may therefore represent an inherent feature of how expectations toward emerging therapies are formed in undergraduate veterinary education.

Despite these limitations, the study has several strengths. It addresses a novel and underexplored topic in veterinary education and clinical psychology, focusing on cognitive–attitudinal factors associated with expectations toward emerging therapies. The inclusion of sociodemographic, educational, experiential, and psychological variables allows for an integrated analytical framework that captures multiple levels of association. Moreover, the use of both bivariate and multivariate approaches provides a robust assessment of the relationships between the independent variables and treatment expectations, supporting the development of a conceptual framework that may guide future research and educational interventions in veterinary medicine.

## 5 Conclusion

Veterinary students’ expectations toward psychedelic-assisted therapies were more closely associated with attitudes and perceived risk than with sociodemographic or academic variables. These cognitive–attitudinal factors were consistently associated with the relationship between background characteristics and expectations of benefit and harm. Overall, expectations were associated with a consistent cognitive–attitudinal framework in which positive attitudes showed positive associations with perceived benefits and inverse associations with perceived risks, while risk perception showed the opposite pattern of associations. These findings suggest that expectations toward emerging therapies in veterinary medicine may be better understood within a broader cognitive–attitudinal framework in which knowledge represents only one component. Future longitudinal studies are needed to establish the potentially causal relationships among these factors and to evaluate how veterinary education may influence their development. Taken together, these findings should be considered exploratory and hypothesis-generating, providing a conceptual framework for future research rather than definitive evidence regarding the cognitive–attitudinal correlates of treatment expectations toward emerging therapies in veterinary medicine.

## Data Availability

The original contributions presented in the study are included in the article/Supplementary material, further inquiries can be directed to the corresponding author.
