# Supplementary material for: Understanding treatment expectations toward emerging therapies in veterinary medicine: the role of knowledge, attitudes, and beliefs toward psychedelic-assisted interventions
Source: Front Vet Sci. 2026 Jul 28;13:1864343. doi: 10.3389/fvets.2026.1864343 (PMC13457715; doi:10.3389/fvets.2026.1864343)
Supplement: Supplementary file 1 [file Data_Sheet_1.DOCX]

**Appendix A. Treatment Expectation Questionnaire (TEX-Q) – Spanish and English versions**

**Instruction:**

Participants rated each item on a 10-point Likert scale ranging from 0 (“not at all”) to 10 (“extremely”).

**A1. Spanish version**

*(Adapted version for veterinary context)*

1. ¿Cuánto alivio en los síntomas de ansiedad espera observar con el tratamiento?

2. ¿Cuánto beneficio cree que tendrán los animales tratados con este tratamiento?

3. ¿Cuánto espera que mejore el bienestar animal con el tratamiento?

4. ¿Cómo de significativa espera que sea la mejora en el bienestar y comportamiento diario de los animales?

5. ¿Cuánto espera que el tratamiento mejore la calidad de vida del paciente tratado?

6. ¿Cuánto espera que el tratamiento mejore la capacidad del paciente para adaptarse a las rutinas y exigencias de su entorno?

7. ¿En qué medida espera que el tratamiento implique riesgos para el perro?

8. ¿Cuánto malestar espera que el tratamiento cause al animal tratado con esta terapia?

9. ¿En qué medida espera que el tratamiento cause efectos secundarios u otros efectos no deseados en el perro?

10. ¿En qué medida espera que el tratamiento reduzca la calidad de vida del paciente?

11. ¿En qué medida espera que el tratamiento limite la capacidad del perro para llevar a cabo sus actividades diarias?

12. ¿En qué medida espera que el procedimiento del tratamiento sea sencillo de aplicar?

13. ¿Hasta qué punto espera estar satisfecho con el procedimiento o proceso del tratamiento?

14. ¿Hasta qué punto espera ser responsable del éxito del tratamiento?

15. ¿Hasta qué punto espera que su propio comportamiento como veterinario influya en el éxito del tratamiento?

**A2. English version**

*(Original/adapted version used for reporting purposes)*

1. How much relief in anxiety symptoms do you expect to observe with the treatment?

2. To what extent do you believe the treated animals will benefit from this treatment?

3. How much improvement in animal welfare do you expect with the treatment?

4. How significant do you expect the improvement to be in the animals’ welfare and daily behavior?

5. To what extent do you expect the treatment to improve the animals’ quality of life?

6. How much do you expect the treatment to improve the patient’s ability to adapt to environmental routines and demands?

7. To what extent do you expect the treatment to involve risks for the dogs?

8. How much discomfort do you expect the treatment to cause the treated animal?

9. To what extent do you expect the treatment to cause side effects or other undesirable effects in the dog?

10. To what extent do you expect the treatment to reduce the dog’s quality of life?

11. To what extent do you expect the treatment to limit the dog’s ability to carry out daily activities?

12. To what extent do you expect the treatment procedure to be easy to administer?

13. How satisfied do you expect to be with the procedure or treatment process?

14. To what extent do you expect to be responsible for the success of the treatment?

15. To what extent do you expect your own behaviour as a veterinarian to influence the success of the treatment?
