# Supplementary material for: Understanding treatment expectations toward emerging therapies in veterinary medicine: the role of knowledge, attitudes, and beliefs toward psychedelic-assisted interventions
Source: Front Vet Sci. 2026 Jul 28;13:1864343. doi: 10.3389/fvets.2026.1864343 (PMC13457715; doi:10.3389/fvets.2026.1864343)
Supplement: Supplementary file 2 [file Table_1.DOCX]

| **Supplementary Table 1**. Frequency distribution (n, %) of responses to the attitudes and beliefs questionnaire on psychedelic-assisted therapies (n = 87). Responses were measured on a 5-point Likert scale (1 = strongly disagree, 5 = strongly agree). | | | | | | |
| --- | --- | --- | --- | --- | --- | --- |
| Item | Strongly  disagree | Disagree | Neither agree  nor disagree | Agree | Strongly  agree | Mean ± SD |
| I1 | 17 (19.5) | 24 (27.6) | 26 (29.9) | 17 (19.5) | 3 (3.4) | 2.6 ± 1.1 |
| I2 | 2 (2.3) | 2 (2.3) | 16 (18.4) | 45 (51.7) | 22 (25.3) | 3.9 ± 0.9 |
| I3 | 1 (1.1) | 1 (1.1) | 4 (4.6) | 55 (63.2) | 26 (29.9) | 4.2 ± 0.7 |
| I4 | 3 (3.4) | 10 (11.5) | 23 (26.4) | 24 (27.6) | 27 (31.0) | 3.7 ± 1.1 |
| I5 | 28 (32.2) | 36 (41.4) | 17 (19.5) | 5 (5.7) | 1 (1.1) | 2.0 ± 0.9 |
| I6 | 2 (2.3) | 0 | 41 (47.7) | 35 (40.2) | 9 (10.3) | 3.6 ± 0.8 |
| I7 | 2 (2.3) | 3 (3.4) | 27 (31.0) | 45 (51.4) | 10 (11.5) | 3.7 ± 0.8 |
| I8 | 2 (2.3) | 3 (3.4) | 6 (6.9) | 35 (40.2) | 41 (47.1) | 4.3 ± 0.9 |
| I1, I consider myself well-informed about psychedelics.  I2, The use of psychedelics increases the risk for subsequent psychiatric disorders.  I3, The use of psychedelics increases the risk of long-term cognitive impairment.  I4, Recreational use of psychedelics should remain illegal.  I5, The use of psychedelics is unsafe even under medical supervision.  I6, Psychedelic use has shown promising results in the treatment of psychiatric disorders.  I7, Psychedelic use may enhance treatment outcomes when used in conjunction with psychotherapy.  I8, Psychedelic use warrants further investigation as a potential treatment for psychiatric disorders. | | | | | | |
