# Supplementary material for: Understanding treatment expectations toward emerging therapies in veterinary medicine: the role of knowledge, attitudes, and beliefs toward psychedelic-assisted interventions
Source: Front Vet Sci. 2026 Jul 28;13:1864343. doi: 10.3389/fvets.2026.1864343 (PMC13457715; doi:10.3389/fvets.2026.1864343)
Supplement: Supplementary file 3 [file Table_2.DOCX]

| **Supplementary Table 2.** Differences in attitudinal and belief-related items across degree programmes (ANOVA with Tukey HSD post hoc). Data for Medicine and Nursing students have been previously reported in a previous publication (Castellano-Ramírez et al. *PLoS One* **2026**, *21*, e0344698) and are included here for comparative purposes. Only statistically significant items are shown. | | | | | |
| --- | --- | --- | --- | --- | --- |
| Item | Veterinary Medicine  (N = 87) | Medicine  (N = 121) | Nursing  (N = 204) | F(2, 409) | p-value ^1^ |
| I1 | 2.6 ± 1.1 ^a^ | 2.9 ± 1.1 ^a^ | 2.2 ± 1.0 ^b^ | 14.6 | <0.001 |
| I5 | 2.0 ± 0.9 ^a^ | 2.7 ± 1.0 ^b^ | 2.8 ± 0.9 ^b^ | 21.4 | <0.001 |
| I6 | 3.6 ± 0.8 ^a^ | 3.3 ± 0.7 ^b^ | 3.2 ± 0.6 ^b^ | 10.9 | <0.001 |
| I7 | 3.7 ± 0.8 ^a^ | 3.6 ± 0.8 ^a^ | 3.3 ± 0.7 ^b^ | 6.5 | 0.002 |
| I8 | 4.3 ± 0.9 ^a^ | 4.1 ± 0.9 ^ab^ | 3.9 ± 0.8 ^b^ | 5.3 | 0.006 |
| ^1^ One-way ANOVA.  Different superscript letters indicate statistically significant differences between groups (Tukey’s HSD, p < 0.05).  I1. I believe I am well-informed about psychedelics.  I5. The use of psychedelics is not safe, even under medical supervision.  I6. Psychedelic use has shown promising results in the treatment of psychiatric disorders.  I7. Psychedelic use may enhance treatment outcomes when used in conjunction with psychotherapy.  I8. Psychedelic use warrants further investigation as a potential treatment for psychiatric disorders. | | | | | |
